# Efficacy of modified EDAS combined with a superficial temporal fascia attachment-dural reversal surgery for the precise treatment of ischemic cerebrovascular disease

**DOI:** 10.3389/fsurg.2023.1087311

**Published:** 2023-03-29

**Authors:** Hanati Nuerlanbieke, Ailiyaer Niyazi, Qinfen Wu, Yang Yuan, Zanghaer Habudele, Xiaoyi Dun, RuRui Wei, Abudula Aisha

**Affiliations:** ^1^ Department of Neurosurgery, The 2th Affiliated Hospital of Xinjiang Medical University, Urumqi, China; ^2^ Xinjiang Key Laboratory of Neurological Disorder Research, Urumqi, China; ^3^ Xinjiang Medical University, Urumqi, China

**Keywords:** ischemic cerebrovascular disease, modified EDAS, revascularization, neovascularization, prognosis

## Abstract

**Objective:**

To investigate the potential therapeutic benefits of Modified EDAS combined with superficial temporal fascia attachment-dural reversal surgery for the treatment of ischemic cerebrovascular disease.

**Methods:**

Retrospective analysis was made on the clinical data of 33 patients with ischemic cerebrovascular disease, who were admitted to the Neurological Diagnosis and Treatment Center of the Second Affiliated Hospital of Xinjiang Medical University from December 2019 to June 2021. All patients were treated with Modified EDAS combined with superficial temporal fascia attachment-dural reversal surgery. At 3 months after operation, the outpatient department rechecked the patient's head CT perfusion imaging (CTP) to understand the intracranial cerebral blood flow perfusion. The DSA of the patient's head was re-examined 6 months after operation to observe the establishment of collateral circulation. The improved Rankin Rating Scale (mRS) score was used to evaluate the good prognosis rate of patients at 6 months after surgery. The mRS score ≤2 was defined as good prognosis.

**Results:**

The preoperative cerebral blood flow (CBF), local blood flow peak time (rTTP), and local mean transit time (rMTT) of 33 patients were 28.235 ml/(100 g·min), 17.702 s, 9.796 s, respectively. At 3 months after surgery, CBF, rTTP, and rMTT were 33.743 ml/(100 g·min), 15.688, and 8.100 s, respectively, with significant differences (*P *< 0.05). At 6 months after operation, the establishment of extracranial and extracranial collateral circulation was observed in all patients by re-examination of head DSA. At 6 months after operation, the good prognosis rate was 81.8%.

**Conclusion:**

The Modified EDAS combined with superficial temporal fascia attachment-dural reversal surgery is safe and effective in the treatment of ischemic cerebrovascular disease, which can significantly increase the establishment of collateral circulation in the operation area and improve the prognosis of patients.

## Introduction

With rapid advances in medical and imaging technology, more and more ischemic cerebrovascular diseases are being diagnosed and treated in a timely manner, and patients’ prognosis are improving, but currently endovascular treatment seems powerless in facing large vessel occlusions, with limited treatment options for patients with complete intracranial arterial occlusions, and intensive medical management or interventions such as angioplasty and stenting failing to improve prognosis (1). For ischemic cerebrovascular disease, surgical treatment including direct and indirect revascularization has become one of the most important therapies in the treatment of ischemic cerebrovascular disease (2). Indirect revascularization is less difficult and challenging than direct revascularization and avoids the risk of occlusion of the bypass vessel. In addition, overperfusion is avoided because there is no dramatic increase in blood flow due to direct anastomosis (3, 4). Indirect bypass surgery is one of the most effective forms of treatment for ischemic cerebrovascular disease, using a variety of tissues as a source of blood supply to induce angiogenesis (5–7), and is widely recognized for its efficacy in improving cerebral perfusion (8–10). Modified EDAS (encephalo-duro-arterio-synangiosis) is an indirect revascularization method, which is widely used in patients with hemodynamic disorders and poor results of medical treatment. Therefore, the authors improved the traditional modified EDAS operation by flipping the superficial temporal artery with fascia 180° and combined with superficial temporal fascial attachment-dural reversal surgery to treat ischemic cerebrovascular patients admitted to the Neurological Treatment Center of the Second Affiliated Hospital of Xinjiang Medical University in recent years, and achieved satisfactory results.

## Materials and methods

### General information

The clinical datas of 33 patients with ischemic cerebrovascular disease, admitted to the Neurological Diagnosis and Treatment Center of the Second Affiliated Hospital of Xinjiang Medical University from December 2019 to June 2021, were retrospectively analyzed. All patients were treated with Modified EDAS combined with superficial temporal fascia attachment-dural reversal surgery, including 25 males and 8 females; The average age was 50 years, ranging from 33 to 71 years. All patients suffered from recurrent dizziness and limb weakness. Among them, 1 case was accompanied by severe vision loss and only light perception, the remaining 32 patients had different degrees of neurological symptoms; mainly limb symptoms, and accompanied by different degrees of abnormal language function. Among the 24 patients, there were 14 patients with type 2 diabetes, 16 patients with hyperlipidemia (3 patients with diabetes), and 6 patients with hyperhomocysteinemia.

### Surgical testimony

1. According to DSA examination, the diagnosis was moyamoya disease, internal carotid artery occlusion or middle cerebral artery occlusion.
2. Cerebral CT perfusion imaging clearly showed the presence of hypoperfusion area;
3. According to DSA examination, Patients with poor collateral circulation (grade 0–2) were assessed using the American Society of Interventional and Therapeutic Neuroradiology/Society of Interventional Radiology (ASITN/SIR) scale;
4. Patients with worsening symptoms or recurrent symptoms after intensive medical treatment.

Contraindications for surgery: (1) Patients with previous intracranial hemorrhage or space occupation; (2) Patients without symptoms of ischemia; (3) Patients with large cerebral infarction; Surgical evidence and contraindications have been added in the paper.

### Imaging data

All 33 patients were examined by CT perfusion imaging (CTP) and DSA. There were ischemic foci in CTP, including 14 cases of temporal lobe ischemia, 10 cases of frontotemporal lobe ischemia, 7 cases of frontotemporal occipital ischemia, and 2 cases of temporal occipital ischemia. The cerebral blood flow (CBF), peak time of regional blood flow (rTTP), regional mean transit time (rMTT) of the lesion area and the mirror image area were obtained by two radiologists with reference to the drawing method of the region of interest (ROI) and recorded. The results of DSA showed that among 33 patients, 9 were suffered from moyamoya disease, 13 were internal carotid artery occlusion, 10 were middle cerebral artery occlusion, and 1 was middle cerebral artery occlusion with moyamoya like changes.

### Surgical method

**Figure 1:**
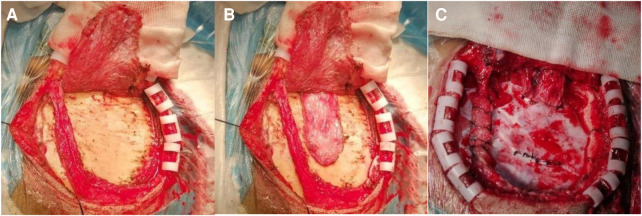
Intraoperative image of a typical patient. (**A**) Free superficial temporal artery with a little fascia; (**B**) dissociating the superficial temporal fascia above the temporalis; (**C**) the superficial temporal fascia was sutured to the dura by crossing under the dural bridge, and the temporal cut dura was turned over and applied to the brain surface.

Transcranial Doppler ultrasound (TCD) was used to locate and mark the superficial temporal artery before operation, and the incision was designed. The superficial temporal artery was microscopically dissociated with approximately 0.5 cm of fascia on both sides, and the superficial temporal fascia was dissociated from the temporalis muscle (Figures 1A,B). The bone flap of about 5 cm × 5 cm was cut by milling cutter, and the incision was designed along the dura vessels. The superficial temporal artery with fascia was turned 180°, and the arachnoid was opened so that the superficial temporal artery was attached to the surface of the brain, where there were blood vessels. Then, the temporalis muscle was separated from the skull surface, and the base was cut laterally (it was appropriate to accommodate the passage of the fascia flap), and the superficial temporal fascial flap was passed through the incision for reservation. The middle meningeal artery was protected and divided into parietal and temporal parts with the middle meningeal artery as the dividing line. A 2–3 cm straight incision was made at the top dura, and a 2 cm × 3 cm arc window was made at the temporal dura. The dura was cut and folded and applied to the surface of the temporal lobe. The superficial temporal fascia passed through the proximal meningeal incision and was tightly sutured to the top dura incision (Figure 1C). The superficial temporal artery entered the cranium from the proximal bone foramen and exited the cranium from the distal bone foramen. A small bone window was opened at the lower edge of the bone flap to accommodate the passage of superficial temporal fascia. During the operation, TCD was used to explore the superficial temporal artery and close the cranium after patency.

### Efficacy evaluation and follow-up

The cranial CT was done on the 1st and 3rd day after operation and before discharge. If the patient has neurological deficit during hospitalization, the cranial CT should be dynamically carried out to determine whether there are new ischemic foci and bleeding. All patients were followed up in the outpatient department at 3 months after operation to understand the cerebral blood flow.

To evaluate the efficacy of neoangiogenesis and guide subsequent treatment management, we asked each patient to follow up for cerebral arteriography at 6 months, 1 year, and the following year. This study was graded according to the method of Matsushima et al. for grading the development of collateral circulation in EDAS (11).

**Figure 2:**
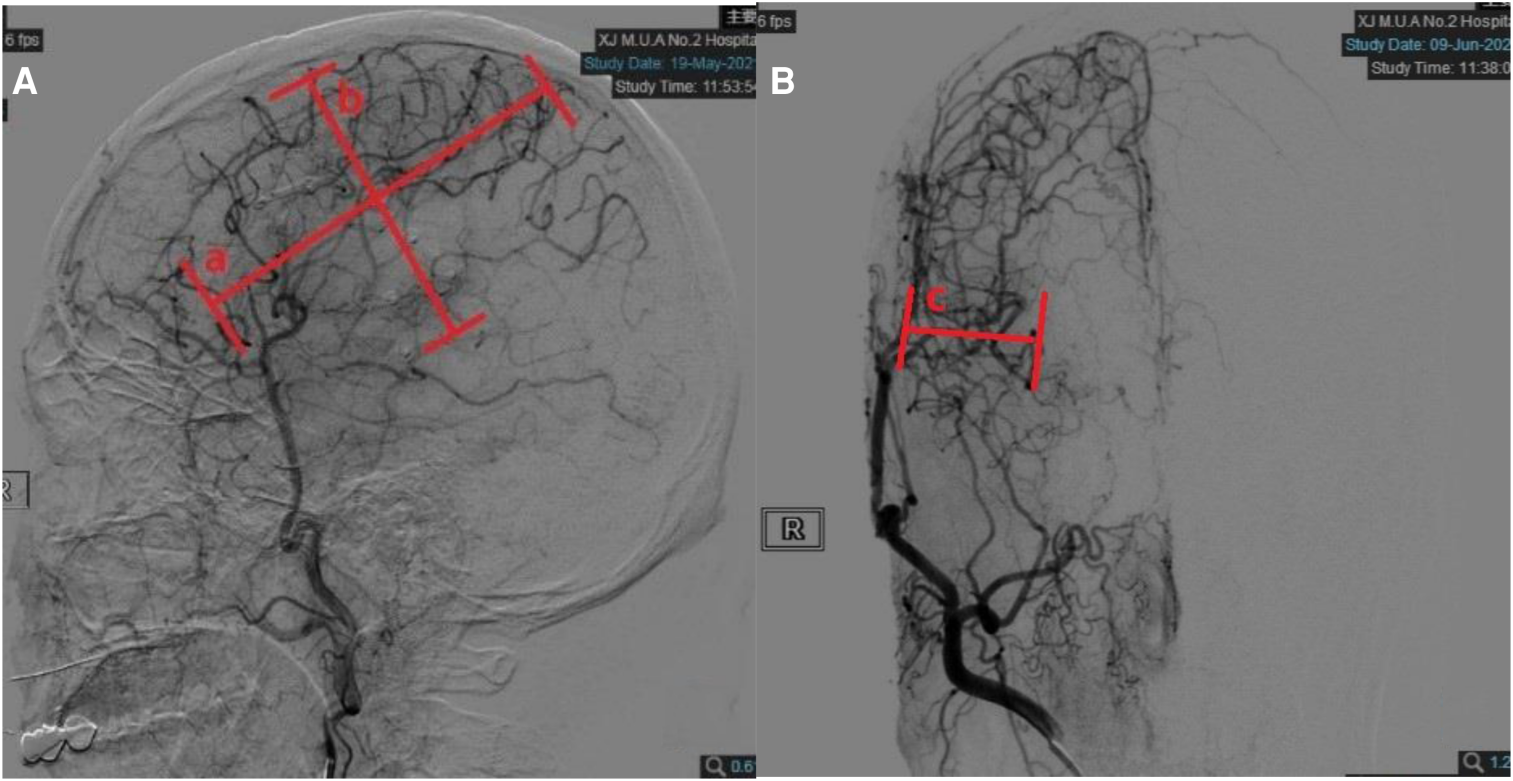
Quantitative measurements on follow-up digital subtraction angiography (DSA). (**A**) Measurements taken on the lateral view. Width (line a) was measured as the longest horizontal distance of the area covered by neoangiogenesis. Height (line b) was measured as the longest vertical distance of the area covered by neoangiogenesis. (**B**) Measurements taken on the anteroposterior view. Depth (line c) was measured as the distance between the furthest end of neoangiogenesis and cortical surface in the temporal region.

Evaluation of anteroposterior neovascularization was worked out by digital subtraction angiography (DSA). The degree of coverage of the cerebral cortex by neovascularization was divided into 4 levels. Level 0:few neovascularizations do not penetrate the cerebral cortex; Level 1:neovascularization covers less than one-third of the hemispheric cortex; Level 2:neovascularization covers more than one-third but less than two-thirds of the hemispheric cortex; Level 3:rich in neovascularization, covering more than two-thirds of the hemispheric cortex (Figure 2).

The patients were followed up for 6 months in the outpatient department or by telephone. The prognosis of the patients at 3 and 6 months after operation was evaluated by the modified Rankin Scale (mRS). mRS score ≤2 was defined as good prognosis.

### Statistical analysis

All data were collected and organized by using excel 2019 software, and SPSS 25.0 software was used for statistical analysis. The mean and standard deviation $\left(\bar{x}\pm s\right)$ were used for the statistical description of CT perfusion imaging parameters and mRS score, and the comparison of CT perfusion imaging parameters 3 months before and after surgery was performed by paired *t*-test (Table 1).

**Table 1:** Comparison of preoperative and postoperative CT perfusion imaging parameters and mRS scores (*X¯* ± *S*, *n* = 33).

Time	CBF [ml/(100 g·min)]	TTP (s)	MTT (s)
Preoperative	28.235 ± 9.665	17.702 ± 3.240	9.796 ± 4.091
3 months after operation	33.743 ± 10.523	15.688 ± 3.370	8.100 ± 3.348
*t*/*F*	−4.130	5.637	3.033
*P*	<0.001	<0.001	<0.005

## Results

### Postoperative condition

The operation was successfully completed in all patients. On the 4th day after the operation, one patient had intracranial hemorrhage, mainly in the temporal cortex. After the superficial temporal artery was protected, the intracranial hematoma was removed. The patient recovered well after the operation. Follow-up head CT scan indicated that intracranial hematoma was basically removed and absorbed. There was no hemiplegia and neurological damage. It was considered that the hemorrhage was caused by the large fluctuation of blood pressure after the occlusion of the middle cerebral artery and had nothing to do with the operation. One patient had epilepsy and convulsions three times after operation, and the symptoms were well controlled after the administration of anti-epileptic drugs. No seizures occurred during the follow-up period. Three patients had new ischemic foci in the operation area after operation, and motor aphasia accompanied by decreased limb muscle strength. They were prescribed medicines and rehabilitation treatments. After 3 months of follow-up, the symptoms were basically improved.

### Changes of blood perfusion (CBF)

**Figure 3:**
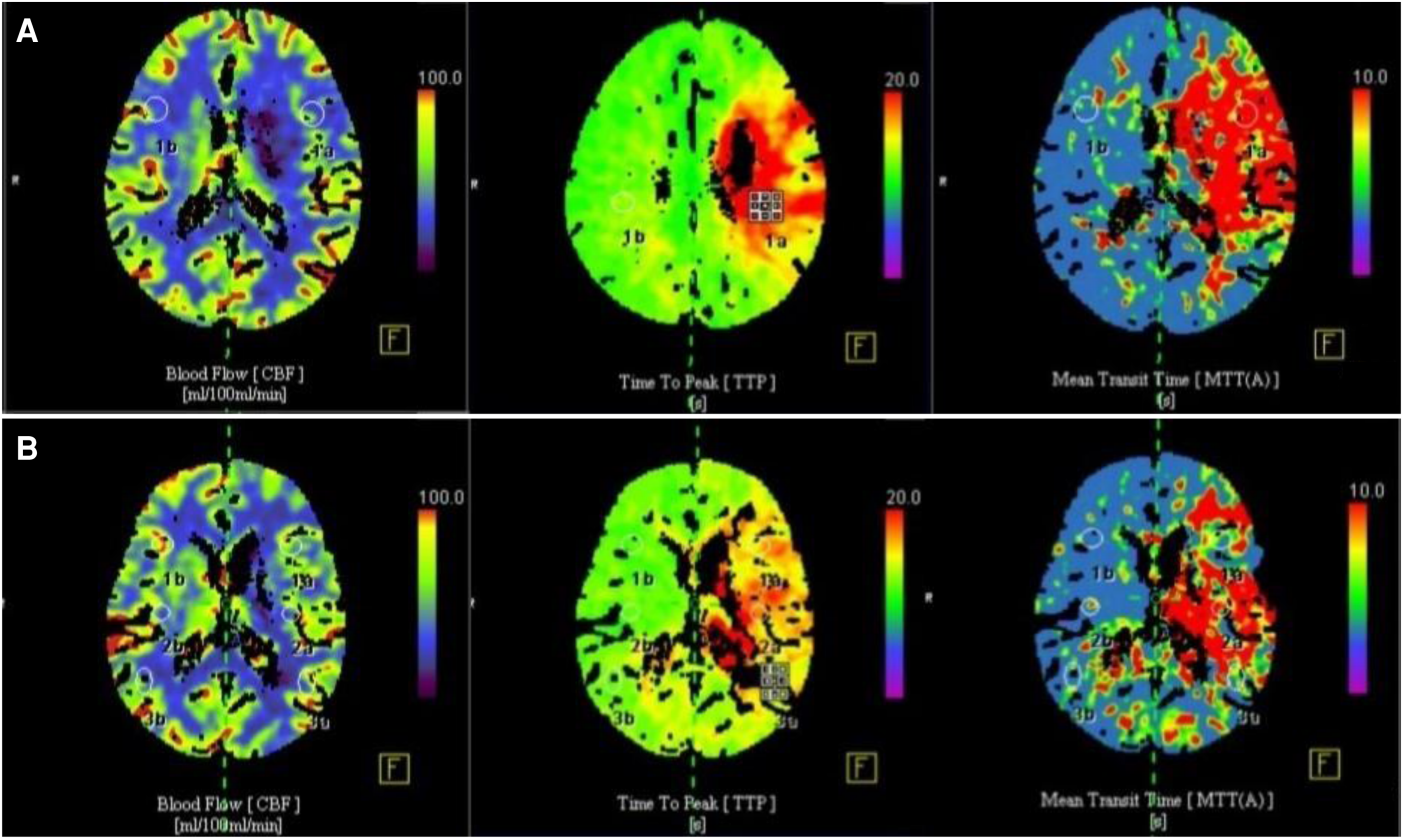
Comparison of CT cerebral perfusion parameters before and 3 months after surgery. (**A**) Preoperative cerebral blood flow was significantly reduced, CBF in cerebral perfusion CTP was significantly reduced, TTP and MTT were prolonged; (**B**) 3 months after operation, cerebral blood flow was significantly improved, CBF in cerebral perfusion CTP was increased, TTP and MTT time were significantly shortened.

In all 33 patients, CBF was significantly increased in cerebral perfusion CTP at 3 months after operation (Figure 3). The difference was statistically significant (*P *< 0.05). rTTP and rMTT were significantly decreased, and the differences were statistically significant compared with those before operation (*P *< 0.05) (Table 1).

### Cranial DSA results

**Figure 4:**
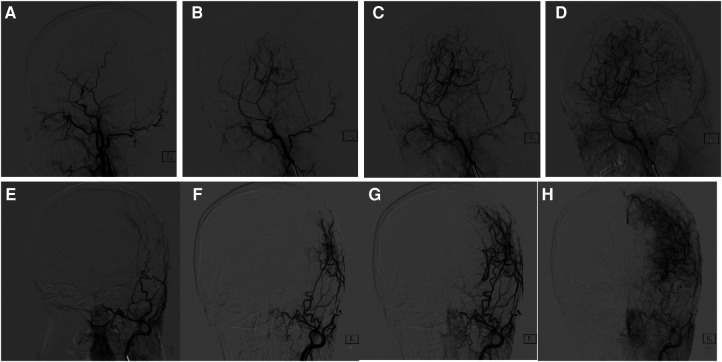
Comparison of cranial DSA before and 6 months after surgery. (**A**) 47-year-old woman with moyamoya disease accepted surgery. (**A**) Lateral view of external carotid artery before surgery. (**B–D**) 6-months follow-up. (**E**) Anteroposterior view of external carotid artery before surgery, (**F–H**) 6-months follow-up. Postoperative DSA showed the establishment of collateral circulation and obvious capillary neogenesis, which were connected with the intracranial.

At 6 months after operation, cranial DSA showed the establishment of the lateral intracranial branch circulation in all patients. Among the 33 patients, 13 (39%) patients achieved Matsushima level 3, 14 (42%) patients achieved Matsushima level 2, and 6 (18%) patients achieved Matsushima level 1. The imaging data of typical patients before and after surgery are shown in Figure 4.

### Patient prognosis

In 33 patients, 3 cases had no effect of operation,1 patient failed to improve his visual acuity due to the large area of occipital lobe ischemia and the damage of visual center; The symptoms of the lower limbs of the affected side were improved in 2 cases. The limb function and language function of the other 30 patients were better than those before operation. The number of patients with good prognosis at 3 and 6 months after operation was 24 and 27, respectively. The good prognosis rate at 6 months was 81.8%.

## Discussion

Revascularization is the surgical establishment of new cerebral blood circulation to improve intracranial ischemia. The method is to use extracranial vessels and blood flow as a donor to divert to the ischemic brain tissue, thus increasing cerebral blood flow to the ischemic area and improving the blood reservation capacity of cerebral vessels (12). Direct revascularization in revascularization is mostly anastomosis of the superficial temporal artery and middle cerebral artery, indirect revascularization includes EMS (encephalo-myo-synangiosis), EDAMS (encephalo-duro-arterio-myo-synangiosis), EDAS (encephalo-duro-arterio-synangiosis), such procedures can significantly improve blood flow, and the prognosis of EDAS can reach 82.4%–86.4% (13). Previous studies have confirmed that modified EDAS procedures can establish effective collateral circulation and improve neuronal cell survival in patients with ischemic cerebrovascular disease. However, indirect revascularization also has disadvantages, as collateral vessel formation requires 3–4 months after surgery to obtain clinical and angiographic results (14, 15). Therefore, in this study, the follow-up was scheduled at 3 and 6 months after surgery, In this study, 33 patients were treated with Modified EDAS combined with superficial temporal fascial attachment-dural reversal surgery. Clinical symptoms improved significantly in almost all patients after surgery. mRS score ≤2 were found in 27 patients after 6 months, with a good prognosis of 81.8%.

The traditional method of EDSA is to suture and paste the superficial temporal artery and dura mater on the brain surface, which is simple and has few complications. Its effectiveness has been confirmed in previous studies. In this study, the modified EDAS combined with superficial temporal fascia attachment-dura reversal surgery differs from traditional EDAS surgery in that the superficial temporal artery-superficial temporal fascia is flipped 180° so that the superficial temporal artery can be attached directly to the surface of the brain and it is easier to establish collateral circulation. In addition, according to the size and location of ischemic position shown in the preoperative imaging data, dura flipped and attached to the temporal lobe can more accurately expand the scope of collateral circulation and improve the temporal lobe blood circulation in patients. Finally, superficial temporal fascial blood vessels are abundant, and fascial tissue with rich blood supply after ensuring the integrity of temporal muscle can promote the regeneration of new blood vessels in the cerebral cortex and improve blood flow to ischemic brain tissue (16).

Precautions for this operation include: (1) The dissection of superficial temporal artery should ensure its integrity to avoid rupture and spasm; (2) The dissociation of superficial temporal fascia should be as complete as possible and not too thick to avoid increasing the incidence of epilepsy; (3) Before operation, it is necessary to examine skull DSA whether the middle meningeal artery intersects with the intracranial for blood supply. When the dura mater is free, the incision should be designed according to the shape of the middle meningeal artery to avoid damaging the middle meningeal artery. In addition, perioperative management plays an important role in enhancing the surgical effect and improving the prognosis of patients. Precautions include: (1) Avoid excessive ventilation. The incidence of cerebral blood flow disorder will be greatly increased under the effect of hypoxia. Therefore, excessive ventilation is not recommended for patients without severe brain edema during operation to avoid the occurrence of new infarction after operation (17). (2) Properly increasing blood pressure during operation can increase blood flow of brain tissue and improve perfusion of ischemic site (18, 19). (3) Postoperative antiepileptic therapy is needed. Epilepsy after operation is one of the main complications, and temporal superficial fascia and temporal muscle contact with cerebral cortex during operation may increase the incidence of epilepsy. Some researchers have confirmed that 18.9% of patients might have seizures on the operation side (20). (4) Statins, edaravone, butylphthalide and other drugs can effectively promote the opening of collateral circulation, which can be used appropriately after surgery (21, 22).

## Conclusion

Although 33 patients in this study benefited from this, many deficiencies still remained, including: (1) the small amount of cases; (2) The postoperative follow-up time is short; (3) Although the treatment of ischemic cerebrovascular disease by dural flip has obtained beneficial results in this study, the relevant literature and the number of cases are still small, and further research and research are needed.
